# A Review of Applications of Metabolomics in Cancer

**DOI:** 10.3390/metabo3030552

**Published:** 2013-07-05

**Authors:** Richard D. Beger

**Affiliations:** National Center for Toxicological Research, US Food and Drug Administration, 3900 NCTR Road, Jefferson, AR 72079, USA; E-Mail: Richard.Beger@fda.hhs.gov; Tel.: +870-543-7080; Fax: +870-543-7686.

**Keywords:** cancer, metabolomics, metabonomics, personalized medicine, biomarker

## Abstract

Cancer is a devastating disease that alters the metabolism of a cell and the surrounding milieu. Metabolomics is a growing and powerful technology capable of detecting hundreds to thousands of metabolites in tissues and biofluids. The recent advances in metabolomics technologies have enabled a deeper investigation into the metabolism of cancer and a better understanding of how cancer cells use glycolysis, known as the “Warburg effect,” advantageously to produce the amino acids, nucleotides and lipids necessary for tumor proliferation and vascularization. Currently, metabolomics research is being used to discover diagnostic cancer biomarkers in the clinic, to better understand its complex heterogeneous nature, to discover pathways involved in cancer that could be used for new targets and to monitor metabolic biomarkers during therapeutic intervention. These metabolomics approaches may also provide clues to personalized cancer treatments by providing useful information to the clinician about the cancer patient’s response to medical interventions.

## 1. Introduction

Metabolomics is the latest of the omics technologies that employs state of the art analytical instrumentation in conjunction with pattern recognition techniques to monitor and discover metabolic changes in subjects related to disease status or in response to a medical or external intervention. Global metabolomics alterations reflect changes due to environmental factors, genetic variation and regulation, changes in gut microflora, and altered kinetic activity or levels of enzymes. Therefore, metabolomics alterations represent changes in the phenotype and molecular physiology [1,2,3]. Metabolomics, like the other omic technologies, is currently being used for the identification of biomarkers and metabolic pathways altered in cancer [4,5,6] and being used to evaluate the efficacy of medical interventions to cancer [7,8,9]. Cancer is a disease that is known to alter cellular metabolism; therefore, metabolomics can play a major role in early detection and diagnosis of cancer and in the evaluation of medical interventions and therapies to cancer [10]. It has been established that aerobic glycolysis increases in cancer and this is known as the “Warburg effect” [11]. Recent advances in analytical technologies and statistical capabilities have provided metabolomics the ability to probe much further into the metabolism of cancer and provide an understanding of how cancer cells use glycolysis advantageously to produce amino acids, nucleotides and lipids necessary for tumor proliferation and vascularization [9,12,13,14,15,16].

Of the omics platforms, metabolomics has great potential to impact clinical health practices due to its ability to rapidly analyze tissue or biofluid samples with little sample preparation; metabolomics provides information that complements the genomic and proteomic profile of a patient. Global metabolic profiling has been referred to as either metabolomics or metabonomics where metabolomics refers to the measurable metabolite pool that exists within a cell or tissue under a particular set of environmental conditions [17] and metabonomics refers to the “quantitative measurement of the dynamic multiparametric metabolic response of living systems to pathophysiological stimuli or genetic modification” [18]. The pool of metabolites detected in biofluids and tissues at a given time will be affected not only by genetic factors but also by lifestyle factors including diet, drugs, exercise, gut microbiota, health-to-disease status, hormonal homeostasis, and age [19,20]. Metabolic profiling is usually referred to as the quantitative study of a group of metabolites that is associated with a particular pathway [21]. Lipidomics is a specialized subset of metabolomics that evaluates lipid profiles [22,23]. Lipids play many important roles in cancer processes including invasion, migration, and proliferation [24].

Another subset of the metabolomics field focuses on using labeled substrates (e.g., ^13^C labeled glucose) to define metabolic fluxes or biomarkers in disease states. This approach enables us to further our understanding of the metabolism in disease or drug responses by following the metabolism of labeled substrates into their pathway products within specific times. For example, glucose can undergo glycolysis to lactate or be shunted through the pentose phosphate pathway to form ribose, and the ^13^C labeled carbons in glucose can reveal how much goes into each pathway. This information provides a better understanding of the pathways that are upregulated or downregulated and can define metabolic phenotypes in disease states [25,26,27,28] or drug response [29,30]. Since glycolysis plays such a major role in cancer, glucose flux technology is ideally suited for understanding cancer and patient response to drug therapy [31].

The ultimate goal of most metabolomics cancer studies is to discover cancer-specific diagnostic, prognostic or predictive biomarkers for a patient. The Food and Drug Administration (FDA). defines a biomarker as a “characteristic that is objectively measured and evaluated as an indicator of normal biological processes, pathogenic processes, or biological responses to a therapeutic intervention” [32]. A diagnostic biomarker is something that can be measured (gene, protein, metabolite, heart rate, tumor size) that indicates patients have a certain disease, while a prognostic biomarker is a measurement that defines the risk for disease occurrence or progression for a patient and a predictive biomarker is a measured characteristic that gives the likelihood that a patient will respond to a particular medical treatment [33]. This review focuses on metabolomics technologies and associated pattern recognition tools that are used to evaluate the metabolome and the metabolomics processes used in biomarker discovery in cancer studies, and the future of metabolomics in cancer research.

## 2. Metabolomics Procedures

**Figure 1:**
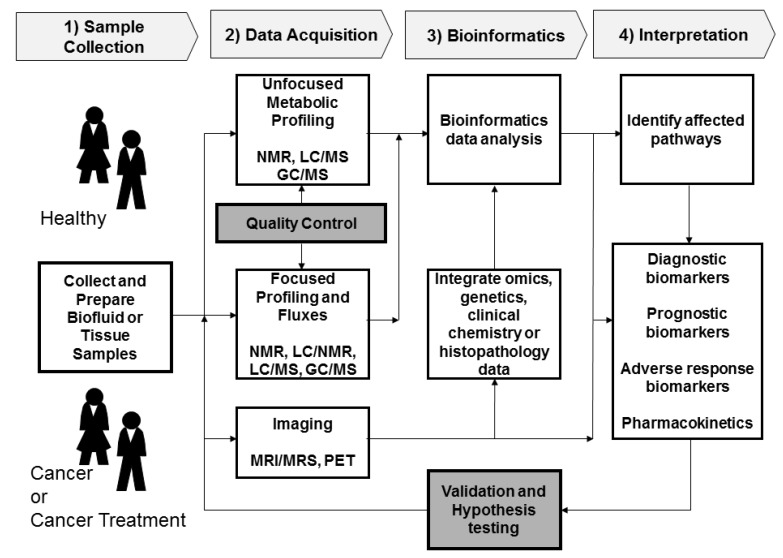
General flow chart of a typical metabolomics experiment in a cancer study.

Figure 1 shows a general flow chart of the logistical steps necessary for conducting cancer metabolomics studies. There are four general steps in planning the metabolomics study: these are sample collection or generation, data acquisition, bioinformatics and interpretation. Once these four steps are completed it is best to form a hypothesis based on the results or test the newly discovered biomarkers in additional studies. Adding quality control during data acquisition is an important step for obtaining reproducible results to assure generation of meaningful metabolomics data. The goal of quality control and standardization is to optimize the reproducibility of the data generated in metabolomics experiments. Inter-laboratory quality standardization of metabolomics approaches will provide additional data for lab-to-lab comparisons. The metabolomics standards initiative (MSI) published minimum reporting standards for metabolomics studies involving *in vivo* samples [34], chemical analysis [35], NMR-based metabolomics [36], and data analysis [37].

### 2.1. Sample Collection

The first step in a planning a metabolomics study involves sample collection, sample storage and sample preparation [38,39]. Urine and blood are typically the samples collected in clinical metabolomics studies but tissue, saliva, breath condensate, cerebral spinal fluid, and pancreatic juices are sample types that have been used in metabolomics studies [6]. A well-designed cancer metabolomics study with multiple time points for sample collection is very important. If the samples are not collected properly or the samples are not stored or processed uniformly, the metabolomics data generated from these samples could be invalid. Therefore sample collection, storage and processing procedures are extremely critical for conducting successful metabolomic studies. There are several steps in sample processing like extractions, use of buffers, and time to processing that need to be planned and followed [40]. Metabolomics studies need to be carefully designed to minimize and account for effects from factors such as gender, age, diet, fasting state, exercise, physical activity, and time of day of sample collection. Capturing patient metadata during sample collection may aid in the interpretation of the results from a metabolomics experiment. Prior to the start of definitive studies, pilot studies from healthy groups should be conducted and documented as part of the study file to demonstrate reproducibility of collection. When possible (such as typical for urine, sera, and plasma), samples should be stored in multiple aliquots right after collection. The use of multiple aliquots is preferred to prevent artifacts generated from multiple freeze/thaw cycles for multiple metabolomics analyses [41]. Tissue samples require snap freezing in liquid nitrogen. Biofluid and tissue samples should be stored at or below −70 °C. Sample processing factors like extraction and pH buffering must be consistent and follow standard operating procedures. For best results during biomarker discovery studies, the dietary diversity in the human population must be minimized which may not be feasible in many clinical studies. If possible, subjects should fast overnight or refrain from food for at least an hour or two before collection of urine or blood samples. In order to account for dietary effects in metabolomics data, a brief description of food consumption over the previous 12–24 h should be included in any clinical or preclinical study. The presence of starvation components increases greatly if subjects are not fed for more than 12 hours. For clinical trials using healthy populations, it is reasonable to request dietary restriction depending on the design and objective of the clinical trial. However, recruitment of best matched controls is still desirable in order to minimize the possibility of variations that arise from gender, age, ethnic origin, and life-style factors like drug and alcohol use. For clinical studies that involve patients with the misfortune of having a severe disease like cancer, it may be considered an undue burden, or unhealthy, to request dietary restriction for minimization of potential diet-related influences on metabolomic profiles. For all clinical studies, independent of dietary restriction, it is desirable to recruit control populations that provide the best controls based on gender, age, and ethnic origin.

### 2.2. Data Acquisition

#### 2.2.1. Sample Analysis

Metabolomics is a sensitive technology capable of detecting metabolic changes due to environmental or physiological stimuli that can occur during a study whether or not they were part of the experimental design. The second step of the metabolomics procedure is the analysis of the biofluid or tissue samples from healthy and cancer patients. If cancer tissue or imaging is obtained and evaluated by metabolomics procedures, it is normally compared to “healthy” tissue adjacent to cancer tissue. Nuclear magnetic resonance (NMR) and mass spectrometric (MS) are often used in combination to evaluate the metabolome by either or both focused or open profiling methods. The advantages and limitations of both NMR and MS techniques have been discussed previously and it has been shown that the two analytical methodologies can provide overlapping yet complementary data [42,43,44]. The combination of multiple techniques on a sample set provides the most powerful method of revealing changes in the metabolome [45]. These changes can be assessed in terms of the molecular pathways being perturbed and allow for the elucidation of the mechanism(s) of disease or toxicity induced by drugs or other agents under a particular set of conditions. In addition, the ability to link metabolites and pathways using these different analytical techniques increases confidence in the identification of potential biomarkers. In addition to analysis of samples, imaging techniques can provide a non-invasive view of the metabolism of cancer and will be discussed later.

#### 2.2.2. Quality Control

During metabolomics data acquisition, quality control is needed to ensure that the data are captured in a reproducible manner and provide meaningful results [41,46,47]. The goal of quality control and standardization is to optimize the reproducibility of the data generated in metabolomics experiments. Inter-laboratory quality standardization of metabolomics approaches will provide additional data for lab-to-lab comparisons. A proper metabolomics study design should include enough samples from each population so that adequate validation and cross-validation can be done, which should reduce the possibilities of false discoveries [48]. Many types of quality control are needed for a successful metabolomics study. For analytical quality control it is optimal to use: (1) 4,4-dimethyl-4-silapentane-1-sulfonic acid (DSS) or 3-(trimethylsilyl)propionic acid (TSP) as a chemical shift standard and a pH standard such as imidazole or difluorotrimethylsilanylphosphonic acid (DFTMP) [49] for NMR.; leucine-enkephalin to ensure the mass accuracy of the mass spectrometer and labeled reference chemical standards for quantitative MS analyses; (2) a synthetic sample of 30–40 representative chemicals for intra-lab quality control during focused and unfocused metabolomics analyses of biofluid and tissue samples; (3) pooled samples from the study that can be used to correct for batch effects in large studies; and (4) pooled human blood standard reference materials (SRM) from NIST that can be used by all metabolomics labs worldwide for inter-lab quality control. In addition, there is a need to determine biomarkers or patterns that are related to the quality of the sample; for example, a sample that is left at room temperature for a long period before analysis, or an urine sample that has bacterial contamination will affect the metabolic profile [50].

### 2.3. Data Processing and Bioinformatics

Once the metabolomics data are collected with quality control measures in place, the third step as shown in Figure 1 is bioinformatics and data analysis. First, the data is processed using vendor software or specialized bioinformatics software for analyzing metabolomics data [51,52,53]. After the metabolomics data are processed and normalized, they can be statistically analyzed. Principal component analysis (PCA) is usually the first type of statistical approach used. PCA is usually applied to the metabolomics data initially to look for patterns related to the end point being studied or to determine if there are any outliers or easily discernible biomarkers. After PCA, many other types of supervised data analysis methods like partial least squares-discriminate analysis (PLS-DA), artificial neural networks (ANN) and other statistical methods can be employed for further data mining in the search for biomarkers [54,55]. The supervised models can be connected to cancer histopathology scores, clinical outcomes, or other omics data to drive the biomarker discovery process. It is essential that the supervised models be tested with external test sets or rigorous internal cross-validated tests using accepted bioinformatics modeling practices so that models and associated biomarkers can be trusted and to limit over fitting of the data [48]. Once models from open profiling data are made and potential spectral features identified as biomarkers, positive identification of the unknown spectral biomarkers is attempted. Identifying the unknown biomarkers in open profiling metabolomics is the most challenging part of metabolomics because the identity of many spectral peaks is unknown. In-house spectral databases and public metabolomics databases like the human metabolome database (HMDB) [56,57], Golm database [58], METLIN database [59], LIPID MAPS [60] and other spectral databases can be used to help identify peaks. Many times a MS or NMR peak will not be identified in the private or public databases and then the peak may be reported as an unknown or determined with the use of standards and additional analytical analyses. Once the metabolomics biomarkers are determined, additional experiments should be done to test or validate the biomarkers. This was just a brief bioinformatics overview and a full review of chemometrics and bioinformatics for cancer metabolomics have been published [55,61].

### 2.4. Interpretation and Validation

The analysis of metabolomics data has provided potential diagnostic or prognostic biomarkers that, in some cases, have been mapped to specific metabolic pathways, processes or transporters. In general, metabolomics open profiling provides a means that can lead to the discovery of new and better biomarkers of cancer and personalized response to cancer therapies. Once potential diagnostic or prognostic cancer biomarkers are found, it is important to follow up these discovery studies with hypothesis driven studies or verification studies of the potential biomarkers. Often times, these hypothesis and validation studies use labeled metabolites, such as ^13^C labeled glucose, to verify that the flux through specific metabolic pathways has occurred in a specified amount of time. Fluxes from ^13^C labeled glucose have been evaluated in pancreatic cancer [26], breast cancer [62] and lung cancer [63] and also to monitor glucose pathways during medical intervention to cancer [14]. If the biomarkers are reproducible, evaluation should then be done at separate metabolomics labs to further evaluate inter-lab variance of the potential biomarker [64]. In follow-up studies it is important to focus on the “context of use” in which the metabolomics biomarker will be evaluated [33,65]. This process is known as biomarker qualification and is intended to establish the utility of a biomarker within a “context of use” such as how sensitive and specific cancer diagnostic or prognostic biomarkers are during drug therapy to cancer. The biomarker or biomarkers under consideration for qualification are expected to be independent of the metabolomics analytical platform used to perform the measurement.

### 2.5. Challenges

The full implementation of metabolomics in the clinic has several challenges. At this time, most clinical metabolomics cancer diagnostic studies have been done on a small population size. Moving from a small population size to a large clinical study will require accepted quality control standards during metabolomics data acquisition and the ability to process and integrate data from multiple instruments at different labs or clinics [41]. The data need to be deposited in large databases where large scale statistical analyses can be done to discover and validate metabolomics biomarkers for cancer. These models and biomarkers may be used to determine whether a cancer patient is responding positively to cancer therapy or if the drug is having little effect or adverse effects. It would be beneficial if the biomarkers for adverse effects of cancer drugs were different than the biomarkers related to radiation poisoning [66,67] and with biomarkers for patients with cachexia [68] or other confounding diseases such as metabolic syndrome.

## 3. Applications of Metabolomics in Cancer Studies

### 3.1. Biomarkers of Cancer

Metabolomics of cancer tissue samples have shown that altered cellular metabolism is a characteristic of almost all cancer types [13,69]. This happens regardless of the organ-specific location of the tumor. Cancer is a complex disease state that changes normal healthy cells into tumor cells primarily using glucose and glutamate to produce energy for cancer cells and to synthesize carbohydrates, fatty acids, amino acids, and nucleotides that are needed for protein synthesis and cellular proliferation [9,70]. These altered metabolic pathways in cancer have been the primary targets for many of the drugs developed for cancer chemotherapy [9]. Therefore, metabolomics can be used to detect altered metabolic pathways in cancer and also could be useful for monitoring cancer drug therapy that targets the altered metabolic pathways. Metabolomics has been evaluated along with standard histopathological analyses of biopsies and many metabolites have been shown to correlate with cancer disease aggressiveness [71].

One of the biggest areas of metabolomics research has been in discovering metabolic biomarkers or patterns of cancer. Cancer has been a fertile ground for metabolomics studies because it is routine to obtain a biopsy of cancer tissue and biofluids from cancer patients while surrounding non-cancerous tissue is sometimes collected. Many different types of samples besides tissue have been used in metabolomics studies of cancer; serum [72,73,74,75,76,77], plasma [78], saliva [79], urine [80,81,82], and breath [83,84] have been used to discover biomarkers of cancer. Serum metabolomics has also been used to assess the stage of pancreatic cancer in a small pilot study [85]. If cancer tissue samples are obtained, they can be evaluated using “high resolution magic angle spinning” (HR MAS) NMR techniques [4] or the aqueous or polar extracts of the cancer tissue and tissue surrounding the cancer tissue can be evaluated by NMR-based and MS-based metabolomics procedures. Both NMR-based and MS-based metabolomics approaches have been used to study cancer.

**Figure 2:**
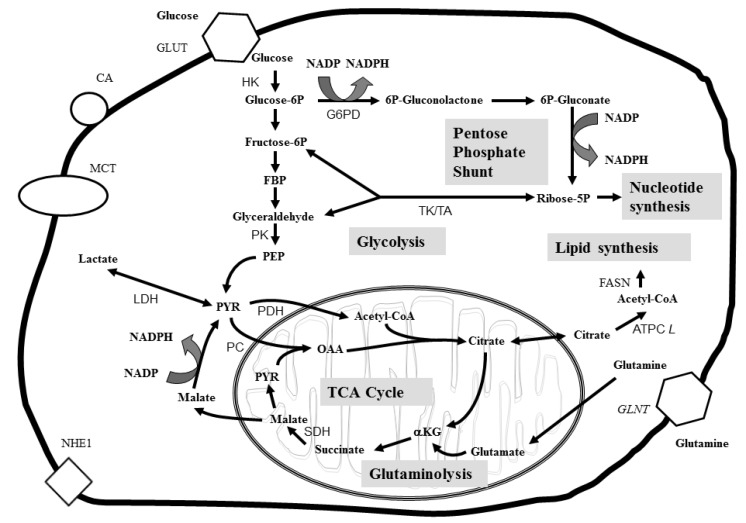
Energy and metabolic pathways and associated protein enzymes and transporters active in cancer. Metabolite abbreviations: αKG, α-ketoglutarate; FBP, fructose 1,6-diphosphate; NADP, nicotinamide adenine dinucleotide phosphate; NADPH, reduced form of nicotinamide adenine dinucleotide phosphate; OAA, oxaloacetate; PEP, phosphoenol pyruvate; PYR, pyruvate. Protein abbreviations: ATPCL, ATP citrate lyase; CA, carbonic anhydrase; FASN, fatty acid synthase; GLUT, glucose transporter; GLNT, glutamine transporter; G6PD, glucose-6-phosphate dehydrogenase; HK, hexokinase; LDH, lactate dehydrogenase; MCT, monocarboxylate transporter; NHE1, Na^+^/H^+^ exchanger; PC, pyruvate carboxylase; PDH, pyruvate dehydrogenase; PK, pyruvate kinase; SDH, succinate dehydrogenase; TK/TA, transketolase/transaldolase.

Figure 2 shows the altered metabolic energy pathways associated with cancer. In general, glycolysis is increased in cancer and is known as the “Warburg” effect [11]. The “Warburg effect” causes tumor cells to import glucose for glycolysis [86]. The main objective of glycolysis is to provide energy for the cancer cell [69], but there is increasing evidence that glycolysis is likely an adaptation to hypoxic conditions of the tumor cell and that glycolysis confers a significant growth advantage by producing the metabolites needed for cancer cells to grow [9,12,15].

Glycolysis is a biochemical process that breaks down glucose and produces two ATP, two NADH and two pyruvate compounds. The fate of pyruvate in cancer cells depends on many factors including oxygen supply and the stage of the cancer tumor growth. Tumor glycolysis metabolism results in an increased acidic environment from the production of lactic acid that is toxic to most cell types but is tolerated by the tumor cell [87]. As lactic acid is released by the tumor, the acidic environment promotes tumor proliferation and invasion to healthy cells through degradation of the extracellular matrix and promotion of angiogenesis [12]. Glucose metabolism through glycolysis also generates the precursors for amino acids, nucleotides, and lipids that are needed for proliferating tumor cells. Glycolysis creates a continuous pool of pyruvate that can be converted to acetyl CoA by pyruvate dehydrogenase (PDH) and acetyl CoA can be used for *de novo* fatty acid synthesis [88]. Increased levels of fatty acids have been observed early in cancer progression, increased in breast cancer [89] and during carcinogenesis [90]. The NADPH that is required for fatty acid synthesis is produced by increased glutaminolysis and up-regulation of the pentose phosphate pathway (PPP) in cancer cells [91,92]. Recent studies have shown that the PPP is up-regulated during glycolysis to produce nucleotides for RNA synthesis [26]. Increased glutaminolysis occurs in proliferating cells like tumor cells [93,94]. Glutamine is an abundant amino acid and glutaminolysis provides tumor cells with another source of energy when glycolysis energy production is not sufficient. In addition, glutamine degradation products are used for anabolic processes. Glucose and glutamine can both be metabolized to nucleic acids, amino acids and lipids that are needed for cell proliferation. 

Besides increased synthesis of amino acids, nucleotides and lipids that are needed for tumor proliferation, other metabolites have been reported as tumor biomarkers in the literature. Alanine is produced by transamination of pyruvate during hypoxic conditions found in liver and brain tumors [95]. Glycine is an essential precursor for purine synthesis that is decreased following hypoxia-inducible factor 1 (HIF-1) signaling [96]. Many metabolic profiling studies have focused on urinary levels of nucleosides as biomarkers of cancer [97] including leukemia [98], breast cancer [99,100,101], colorectal cancer [102], and hepatocellular carcinoma [103] and are detected independent of age or gender of the patient. Modified nucleosides are produced by methylation and oxidative damage to the DNA and both of these processes are known to be related to developing cancer. Modified nucleosides are not reused by cells and ultimately are excreted in the urine. Even though increased urinary levels of modified nucleosides have been detected in numerous cancer studies, the levels detected by different analytical techniques are not consistent from one lab to another. Therefore, there is a need to optimize the analytical techniques to detect nucleosides [97]; the use of widely accepted standardized quality control samples and procedures may help decrease inter-lab variability. During DNA methylation the methyl group on *S*-adenosyl-L-methionine (SAMe) is transferred to DNA by a family of DNA methyltransferase enzyme; in a similar process a methyl group is transferred from SAMe to glycine by glycine N-methyl transferase to produce sacrosine. Increased levels of urinary sarcosine has been observed in prostate cancer patients and were shown to be increased during prostate cancer progression and metastasis [104]. The prostate cancer study also showed that introduction of sarcosine induced a malignant phenotype [104].

Since 1951, fatty acid synthesis has been known to be increased in tumors [105]. Lipids are involved in many tumor processes including cell dislodgement, invasion, migration, and proliferation [24]. Choline, phosphocholine, phosphatidylcholine, and glycerophosphocholine are needed for cell wall synthesis and are increased in brain, breast, prostate and liver cancers [6,106,107,108]. Lysophosphatidylcholines (lysoPCs) are lipid intermediates that can used to form PCs or are the products of phospholipases acting on PCs. Decreased blood levels of lysoPCs have been observed in lung [109] and liver cancer [110] while lysophosphatic acid has been reported as increased in ovarian cancer [111]. Tissue levels of phosphoinositides have been increased in several tumors [112,113] while three phosphatidylinositols in plasma were found to be reduced in pancreatic cancer [78]. Phosphatidylinositols have been reported to be involved with signaling for cancer cell growth motility and proliferation [114]. Sphingolipids have been reported as tissue biomarkers of cancer for their role in cancer growth and proliferation and they have been used in anti-cancer therapy [115]. Sphingosine-1-phosphate is released from cancer and is involved with tumorigenic and angiogenic properties of tumors [114,116,117]. The analyses of lipids have shown that they play a significant role in tumor metabolism, growth, and signaling and will be important components of cancer lipidomics studies.

High resolution magic angle spinning nuclear magnetic spectroscopy (HR-MAS-NMR) spectroscopy has been used to detect metabolic changes in cancer tissue samples [4,118,119]. HR-MAS-NMR it is non-destructive to the tissue and has well-resolved spectra compared to *in vivo* MRS imaging. The spectral resolution quality in MAS-NMR has improved in recent years to the point where HR-MAS-NMR spectra of breast cancer tissues were comparable in quality to the spectra of perchloric acid extracts of cancer tissue [120]. HR-MAS-NMR has been used in open profiling with pattern recognition of spectra and by identifying specific metabolites as diagnostic biomarkers of cancer. An HR-MAS-NMR spectral library was used to find metabolic differences between glial and neural ectodermal tumors in children [121]. In another cancer study, HR-MAS-NMR was used to profile head and neck squamous cell carcinoma (HNSCC) tissue, matched normal adjacent tissue and lymph-node metastatic (LN-Met) tissue [122]. Higher levels of lactate, amino acids, choline and lower levels of triglyceride were found in the HNSCC and LN-MET tissues than adjacent normal tissue. These metabolites are associated with increased glycolysis, anaplerosis, membrane choline metabolism, and altered energy metabolism that are needed by cancer cells to deliver energy and substrates required for rapid cell proliferation. HR-MAS-NMR was used to obtain metabolic profiles of human malignant and non-malignant prostate tissue samples [123]. Metabolic ratios of (glycerophosphocholine and phosphocholine)/creatine, myo-inositol/scyllo-inositol, choline/creatine and other ratios were found to correlate with the number of tumor cells, tumor cell proliferation, and for non-malignant tissue the distance to the nearest tumor and its Gleason score. Gleason scores are determined on two tissue scores assigned by a pathologist to a biopsy or surgical tissue and provide some prognostic value. The first score is based on the tissue pattern it most likely resembles and the second score is the next tissue pattern it most likely resembles. The two pathology scores are added to determine the final Gleason score. The higher the Gleason score (ranges 2–10) the worse the prognosis. In a separate prostate cancer study, MRS was able to differentiate between Gleason scores of 6 and 7 and predict tumor perineural invasion [124]. In colorectal cancer, HR-MAS-NMR and GC/MS were used to determine that metabolites involved with glycolysis, hypoxia, lipid metabolism and inflammation were altered [125]. One advantage of HR-MAS-NMR approaches is that the results can be directly translated to MRS imaging studies. Furthermore, HR-MAS-NMR has been used to aid interpretation of proton MRS imaging of tumors. The translation of *ex vivo* MAS to *in vivo* MRS is a feature that should benefit the clinical use of MRS for cancer diagnosis.

### 3.2. Imaging Metabolic Biomarkers

X-ray, positron-emission tomography (PET), single photon-emission computed tomography (SPECT), magnetic resonance imaging (MRI), magnetic resonance spectroscopy imaging (MRSI), and ultrasound are *in vivo* imaging techniques that have been used to help diagnose cancer [126]. PET is a functional image of tumors that relies on the fact that tumors have increased glucose uptake compared to normal cells. PET uses a radiolabeled glucose analogue, 2-[^18^F]fluoro-2-deoxy-D-glucose (FDG), to detect glucose uptake by the tumor cells [127,128]. MRI and MRSI have been used to non-invasively diagnose and evaluate treatment to many types of cancer [129,130,131,132,133,134]. Dynamic contrast enhanced (DCE) MRI is often done with contrast agents to enhance the image quality for diagnostic purposes [133,135]. Diffusion weighted MRI measures the motion of water in tissue and provides information that is complementary to standard MRI for the detection of oncology-related issues [134]. MRI is often used in combination with mammography to diagnose breast cancer and is much more accurate and sensitive than a mammogram alone [136,137]. MRSI is a technique that collects an NMR spectrum from a small three-dimensional voxel in the tissue. MRSI has advantages over standard MRI in that it can detect many of the metabolic changes in tumors besides glucose uptake and is better than MRI which only detects water density or water movement. MRSI has a disadvantage over MRI in that it has much longer acquisition times, harder to process the data, and lack of familiarity with clinicians. MRSI detection of choline has been used to diagnose and monitor breast [138], brain [139] and prostate cancers [140]. MRSI detection of choline has been reported to be 100% sensitive for detection of malignant versus benign breast tumors and could be used to reduce the need of unnecessary breast biopsies [138]. The ability to use single voxel MRSI data to classify nine different types of brain cancer has been successful [141]. Alusta and coworkers used four steps to process the MRSI data before building a classification modeling that included data normalization, re-calibration of the spectra to specific peaks, weighting of the data, and re-normalization of the MRSI spectral data. The four step data processing increased the accuracy of predicting nine different brain cancer categories from 31% to 95% [141].

### 3.3 Metabolomics and Cancer Drug Therapy

The first medical therapies for cancer used chemicals that were called “antimetabolites” [142]. They were named antimetabolites because they were chemically similar to endogenous metabolites in select pathways and interfered with the normal metabolism in the pathway. Cytarabine, 5-fluorouracil, and methotrexate are antimetabolites that targeted late stage DNA synthesis. There is a lot of interest in targeting altered cancer metabolism as a therapeutic strategy during treatment [9,143,144]. *De novo* fatty acid synthesis is a cancer therapy target and the drug Orlistat and several other drugs target the fatty acid synthase (FASN) enzyme while other cancer drugs target ATP citrate lyase (ACLY). Lonidamine, 2-deoxyglucose, and 3-bromopyruvate are drugs being developed that target the glycolysis enzyme, hexokinase (HK), while another drug under development targets pyruvate kinase (PK). Each of these drugs should inhibit glycolysis, which is known to be upregulated in cancer. It is anticipated that cancer drugs that inhibit upregulated enzymes in cancer cells should reduce endogenous metabolites essential for cancer growth thus reducing cancer cell proliferation and may reduce transformation in pre-cancer cells. The levels of endogenous metabolites should start changing before the tumor reduces size, which is a common clinical end point for cancer therapy. Transporters are another cancer therapy target, where the drug phloretin targets the glucose transporter (GLUT) and the monocarboxylate transporter (MCT). There are several other drugs in clinical development that target MCT and cariporide is a drug in preclinical development that targets the Na^+^/H^+^ exchanger (NHE1). Compared with other omics, metabolomics is best suited to evaluate whether these cancer therapies ultimately cause changes to metabolic pathways while at the same time it can detect drug pharmacokinetics. Weiss [145] described a hypothetical pharmacometabolomics study that showed healthy patients responded in a way that was different from cancer treatment patients. Furthermore, there were metabolic differences in cancer patients that were taking endothelial growth factor inhibitors and those taking Raf inhibitors. Pharmacometabolomics was used to evaluate docetaxel-treated human breast cells and showed a dose-dependent and time-dependent response in phospholipid and glutathione metabolism [146]. In the clinic, pharmacometabonomics has been used to show that the levels of lipids in patients taking capecitabine for inoperable colorectal cancer correlated with the severity of the toxicity; the higher the levels, the greater the severity [147]. In the future, it will be important to couple these pharmacometabolomics clinical cancer studies with other systems biology information, like genetics, miRNA, mRNA and imaging to determine whether the metabolic response can be correlated with cancer grade, adverse events, and whether the cancer is growing or receding. Because pharmacometabolomics has the capability to monitor how patients metabolically respond to drugs, there is a lot of interest in using metabolomics in cancer detection, cancer prognosis and cancer therapy management [148].

## 4. Conclusions

Cancer is a disease that alters the metabolism of a cell and metabolomics approaches are being employed to better understand these changes in cancer metabolism. Both open and focused metabolomics approaches along with imaging techniques can be used to monitor metabolism in cancer for both diagnostic and prognostic biomarkers. The metabolomics biomarkers of cancer listed in the manuscript are just experimental and have not been validated. Some of these metabolomics biomarkers could be submitted to the FDA or Biomarker Consortium [149,150]. Metabolomics has shown a lot of promise for personalized medicine and cancer diagnostics; however, initial results are still experimental and it has to overcome many challenges in order to be fully implemented and reach its full potential. The challenges include globally accepted quality control standards, identification of unknown peaks, validation studies, the ability to differentiate between radiation damage and drug adverse events, addition of clinical metadata to metabolomics, sharing of clinical metabolomics data in the clinic with regulatory agencies, storing and interpreting the clinical metabolomics data, and implementing NMR spectrometers and MS in the clinical environment. Nevertheless, Nicholson and others have shown how metabolomics can be implemented in hospitals to phenotype patients during surgery and critical care [151,152]. Some of the metabolomics analyses need to be done rapidly, in real time for patients undergoing surgery. Recently, real time tissue analysis during surgery has been realized using evaporative ionization MS from the thermal degradation of tissue during electrosurgery that informs the surgeon whether the tumor being cut is malignant tissue [153]. The future of personalized medicine in cancer will use metabolomics to evaluate many parts of cancer patient care including diagnostic biomarkers, metabolic responses from patients taking drugs in terms of efficacy, informing the doctor about gut microbiome changes before, during and after surgery, adverse events, pharmaceutical kinetics and evaluation of prognostic markers to determine risks during surgery and cancer therapy. Metabolomics cancer studies are now being conducted with information about tumor genetics [154], and other systems biology information about the patient such as levels of miRNAs [155,156] proteins [154,157,158], and transcripts [81,154,159] and these combined approaches will become much more common in the future because the information metabolomics provides is downstream and complimentary to the other omics technologies. Metabolomics combined with other systems biology datasets will further our understanding of the complexity of cancer and detect personalized responses to cancer therapy [160]. The altered metabolism in tumors is leading to the development of many cancer drug therapies that target the altered metabolic pathways and associated enzymes or transporters involved in cancer. Therefore, metabolomics analyses of tissue biopsies and biofluids from cancer patients and medical interventions to cancer will continue to be useful in providing a better understanding of the complex nature of cancer and for providing information to the clinician about the patient’s response to medical interventions to cancer. Standardized and accepted quality controls and metabolomics databases to help clinicians understand metabolomics results are still needed for metabolomics to be successful and broadly applied in the clinic setting.

## References

[1] Fiehn O. (2002). Metabolomics—The link between genotypes and phenotypes. Plant Mol. Biol..

[2] Clayton T.A., Lindon J.C., Cloarec O., Antti H., Charuel C., Hanton G., Provost J.P., Le Net J.L., Baker J.D., Walley R.J. (2006). Pharmaco-metabonomic phenotyping and personalized drug treatment. Nature.

[3] Holmes E., Wilson I.D., Nicholson J.K. (2008). Metabolic phenotyping in health and disease. Cell.

[4] Griffin J.L., Shockcor J.P. (2004). Metabolic profiles of cancer cells. Nat. Rev. Cancer.

[5] Kim Y.S., Maruvada P., Milner J.A. (2008). Metabolomics in biomarker discovery: Future uses for cancer prevention. Future Oncol..

[6] Spratlin J.L., Serkova N.J., Eckhardt S.G. (2009). Clinical applications of metabolomics in oncology: A review. Clin. Cancer Res..

[7] Fan T.W., Lane A.N., Higashi R.M. (2004). The promise of metabolomics in cancer molecular therapeutics. Curr. Opin. Mol. Ther..

[8] Chung Y.L., Griffiths J.R. (2007). Using metabolomics to monitor anticancer drugs. Ernst Schering Found. Symp. Proc..

[9] Vander Heiden M.G. (2011). Targeting cancer metabolism: A therapeutic window opens. Nat. Rev. Drug Discov..

[10] Kaddurah-Daouk R., Kristal B.S., Weinshilboum R.M. (2008). Metabolomics: A global biochemical approach to drug response and disease. Annu. Rev. Pharmacol. Toxciol..

[11] Warburg O. (1956). On the origin of cancer cells. Science.

[12] Gatenby R.A., Gillies R.J. (2004). Why do cancers have high aerobic glycolysis. Nat. Rev. Cancer.

[13] Vander Heiden M.G., Cantley L.C., Thompson C.B. (2009). Understanding the warburg effect: The metabolic requirements of cell proliferation. Science.

[14] Vizán P., Sánchez-Tena S., Alcarraz-Vizán G., Soler M., Messeguer R., Pujol M.D., Lee W.-N.P., Cascante M. (2009). Characterization of the metabolic changes underlying growth factor angiogenic activation: Identification of new potential therapeutic targets. Carcinogenesis.

[15] Israel M., Schwartz L. (2011). The metabolic advantage of tumor cells. Mol. Cancer.

[16] Weljie A.M., Jirik F.R. (2011). Hypoxia-induced metabolic shifts in cancer cells: Moving beyond the Warburg effect. Int. J. Biochem. Cell Biol..

[17] Fiehn O. (2001). Combining genomics, metabolome analysis and biochemical modeling to understand metabolic networks. Int. J. Genomics.

[18] Nicholson J.K., Lindon J.C., Holmes E. (1999). Metabonomics: Understanding the metabolic responses of living systems to pathophysiological stimuli via multivariate statistical analysis of biological nmr data. Xenobiotica.

[19] Nicholson J.K., Connelly J., Lindon J.C., Holmes E. (2002). Metabonomics: A platform for studying drug toxicity and gene function. Nat. Rev. Drug Discov..

[20] Nicholson J.K., Holmes E., Wilson I.D. (2005). Gut microorganisms, mammalian metabolism and personalized health care. Nat. Rev. Micro..

[21] Roessner U., Luedemann A., Brust D., Fiehn O., Linke T., Willmitzer L., Fernie A.R. (2001). Metabolic profiling allows comprehensive phenotyping of genetically or environmentally modified plant systems. The Plant Cell.

[22] Han X., Gross R.W. (2003). Global analyses of cellular lipidomes directly from crude extracts of biological samples by esi mass spectrometry: A bridge to lipidomics. J. Lipid Res..

[23] Wenk M.R. (2010). Lipidomics: New tools and applications. Cell.

[24] Fernandis A.Z., Wenk M.R. (2009). Lipid-based biomarkers of cancer. J. Chrom. B.

[25] Boros L.G., Brackett D.J., Harrigan G.G. (2003). Metabolic biomarker and kinase drug target discovery in cancer using stable isotope-based dynamic metabolic profiling (sidmap). Curr. Cancer Drug Targets.

[26] Boros L.G., Lerner M.R., Morgan D.L., Taylor S.L., Smith B.J., Postier R.G., Brackett D.J. (2005). [1,2–13c2]-d-glucose profiles of the serum, liver, pancreas, and dmba-induced pancreatic tumors of rats. Pancreas.

[27] Lane N.L., Fan T.W.-H., Higashi R.M., Tan J., Bousamra M., Miller D.M. (2009). Prospects for clinical cancer metabolomics using stable isotope tracers. Exp. Mol. Pathol..

[28] Zhang G.-F., Sadhukhan S., Tochtrop G.P., Brunengraber H. (2011). Metabolomics, pathway regulation, and pathway discovery. J. Biol. Chem..

[29] Beger R., Hansen D., Schnackenberg L., Cross B., Fatollahi J., Lagunero F.T., Sarnyai Z., Boros L. (2009). Single valproic acid treatment inhibits glycogen and rna ribose turnover while disrupting glucose-derived cholesterol synthesis in liver as revealed by the [u-13C6]-d-glucose tracer in mice. Metabolomics.

[30] Boros L.G., Lee W.-N.P., Cascante M. (2002). Imatinib and chronic-phase leukemias. N. Engl. J. Med..

[31] Boros L.G. (2005). Metabolic targeted therapy of cancer: Current tracer technologies and future drug design strategies in the old metabolic network. Metabolomics.

[32] (2006). Guidance for industry pharmacogenomic data submissions.

[33] Beger R., Colatsky T. (2012). Metabolomics data and the biomarker qualification process. Metabolomics.

[34] Griffin J., Nicholls A., Daykin C., Heald S., Keun H., Schuppe-Koistinen I., Griffiths J., Cheng L., Rocca-Serra P., Rubtsov D. (2007). Standard reporting requirements for biological samples in metabolomics experiments: Mammalian/in vivo experiments. Metabolomics.

[35] Sumner L.W., Amberg A., Barrett D., Beger R., Beale M.H., Daykin C., Fan T.W., Fiehn O., Goodacre R., Griffin J.L. (2007). Proposed minimum reporting standards for chemical analysis. Metabolomics.

[36] Rubtsov D., Jenkins H., Ludwig C., Easton J., Viant M., Günther U., Griffin J., Hardy N. (2007). Proposed reporting requirements for the description of nmr-based metabolomics experiments. Metabolomics.

[37] Goodacre R., Baker D.J., Beger R., Bessant C., Broadhurst D., Connor S., Capuani G., Craig A., Ebbels T., Kell D.B. (2007). Proposed minimum reporting standards for data analysis in metabolomics. Metabolomics.

[38] Ganti S., Weiss R.H. (2011). Urine metabolomics for kidney cancer detection and biomarker discovery. Urol. Oncol..

[39] Mamas M., Dunn W.B., Neyes L., Goodacre R. (2010). The role of metabolites and metabolomics in clinically applicable biomarkers of disease. Arch. Toxicol..

[40] Serkova N., Glunde K. (2009). Metabolomics of cancer. Methods Mol. Biol..

[41] Dunn W.B., Broadhurst D., Begley P., Zelena E., Francis-McIntyre S., Anderson N., Brown M., Knowles J.D., Halsall A., Haselden J.N. (2011). Procedures for large-scale metabolic profiling of serum and plasma using gas chromatography and liquid chromatography coupled to mass spectrometry. Nat. Protocols.

[42] Dunn W.B., Ellis D.I. (2005). Metabolomics: Current analytical platforms and methodologies. Trends Anal. Chem..

[43] Robertson D.G. (2005). Metabonomics in toxicology: A review. Toxicol. Sci..

[44] Lenz E.M., Wilson I.D. (2007). Analytical strategies in metabonomics. J. Prot. Res..

[45] Psychogios N., Hau D., Peng J., Guo A., Mandal R., Bouatra S., Sinelnikov I., Krishnamurthy R., Eisner R., Gautam B. (2011). The human serum metabolome. PLoS One.

[46] Sangster T., Major H., Plumb R., Wilson A.J., Wilson I.D. (2006). A pragmatic and readily implemented quality control strategy for HPLC-MS and GC-MS-based metabonomic analysis. Analyst.

[47] Dunn W.B., Wilson I.D., Nicholls A.W., Broadhurst D. (2012). The importance of experimental design and qc samples in large-scale and ms-driven untargeted metabolomic studies of humans. Bioanalysis.

[48] Broadhurst D., Kell D. (2006). Statistical strategies for avoiding false discoveries in metabolomics and related experiments. Metabolomics.

[49] Reily M.D., Robosky L.C., Manning M.L., Butler A., Baker J.D., Winters R.T. (2006). Dftmp, an NMR reagent for assessing the near-neutral pH of biological samples. J. Am. Chem. Soc..

[50] Saude E., Sykes B. (2007). Urine stability for metabolomic studies: Effects of preparation and storage. Metabolomics.

[51] Katajamaa M., Orešič M. (2007). Data processing for mass spectrometry-based metabolomics. J. Chrom. A.

[52] O'Sullivan A., Avizonis D., German J.B., Slupsky C.M. (2007). Software tools for NMR metabolomics. eMagRes..

[53] Sugimoto M., Kawakami M., Robert M., Soga T., Tomita M. (2012). Bioinformatics tools for mass spectrometry-based metabolomics data processing and analysis. Curr. Bioinformatics.

[54] Fonville J.M., Richards S.E., Barton R.H., Boulange C.L., Ebbels T.M.D., Nicholson J.K., Holmes E., Dumas M.-E. (2010). The evolution of partial least squares models and related chemometric approaches in metabonomics and metabolic phenotyping. J. Chemometrics.

[55] Madsen R., Lundstedt T., Trygg J. (2010). Chemometrics in metabolomics—a review in human disease diagnosis. Anal. Chim. Acta.

[56] Wishart D.S., Knox C., Guo A.C., Eisner R., Young N., Gautam B., Hau D.D., Psychogios N., Dong E., Bouatra S. (2009). Hmdb: A knowledgebase for the human metabolome. Nucl. Acids Res..

[57] Wishart D.S., Tzur D., Knox C., Eisner R., Guo A.C., Young N., Cheng D., Jewell K., Arndt D., Sawhney S. (2007). Hmdb: The human metabolome database. Nucl. Acids Res..

[58] Kopka J., Schauer N., Krueger S., Birkemeyer C., Usadel B., Bergmuller E., Dormann P., Weckwerth W., Gibon Y., Stitt M. (2005). Gmd@csb.Db: The golm metabolome database. Bioinformatics.

[59] Smith C.A., O’Maille G., Want E.J., Qin C., Trauger S.A., Brandon T.R., Custodio D.E., Abagyan R., Siuzdak G. (2005). Metlin—a metabolite mass spectral database. Ther. Drug Monit..

[60] Sud M., Fahy E., Cotter D., Brown A., Dennis E.A., Glass C.K., Merrill A.H., Murphy R.C., Raetz C.R.H., Russell D.W. (2007). Lmsd: Lipid maps structure database. Nucl. Acids Res..

[61] Blekherman G., Laubenbacher R., Cortes D.F., Mendes P., Torti F.M., Akman S., Torti S.V., Shulaev V. (2011). Bioinformatics tools for cancer metabolomics. Metabolomics.

[62] Yang C., Richardson A.D., Smith J.W., Osterman A. (2007). Comparative metabolomics of breast cancer. Pacific Symposium on Biocomputing.

[63] Lane A., Fan T.-M., Bousamra M., Higashi R., Yan J., Miller D. (2011). Stable isotope-resolved metabolomics (sirm) in cancer research with clinical application to nonsmall cell lung cancer. OMICS.

[64] Mamas M., Dunn W., Neyses L., Goodacre R. (2011). The role of metabolites and metabolomics in clinically applicable biomarkers of disease. Arch. Toxicol..

[65] Matheis K., Laurie D., Andriamandroso C., Arber N., Badimon L., Benain X., Bendjama K., Clavier I., Colman P., Firat H. (2011). A generic operational strategy to qualify translational safety biomarkers. Drug Discov. Today.

[66] Johnson C.H., Patterson A.D., Krausz K.W., Lanz C., Kang D.W., Luecke H., Gonzalez F.J., Idle J.R. (2011). Radiation metabolomics. 4. UPLC-ESI-QTOFMS-based metabolomics for urinary biomarker discovery in gamma-irradiated rats. Radiation Res..

[67] Coy S.L., Cheema A.K., Tyburski J.B., Laiakis E.C., Collins S.P., Fornace A.J. (2011). Radiation metabolomics and its potential in biodosimetry. Int. J Rad. Bio..

[68] O’Connell T., Ardeshirpour F., Asher S., Winnike J., Yin X., George J., Guttridge D., He W., Wysong A., Willis M. (2008). Metabolomic analysis of cancer cachexia reveals distinct lipid and glucose alterations. Metabolomics.

[69] Seyfried T., Shelton L. (2010). Cancer as a metabolic disease. Nutr. Metab..

[70] Kim J.-w., Dang C.V. (2006). Cancer's molecular sweet tooth and the Warburg effect. Cancer Res..

[71] Brown M., McDunn J., Gunst P., Smith E., Milburn M., Troyer D., Lawton K. (2012). Cancer detection and biopsy classification using concurrent histopathological and metabolomic analysis of core biopsies. Genome Medicine.

[72] Kobayashi T., Nishiumi S., Ikeda A., Yoshie T., Sakai A., Matsubara A., Izumi Y., Tsumura H., Tsuda M., Nishisaki H. (2013). A novel serum metabolomics-based diagnostic approach to pancreatic cancer. Cancer Epidemiol. Biomarkers Prev..

[73] Ikeda A., Nishiumi S., Shinohara M., Yoshie T., Hatano N., Okuno T., Bamba T., Fukusaki E., Takenawa T., Azuma T. (2012). Serum metabolomics as a novel diagnostic approach for gastrointestinal cancer. Biomed. Chromatogr..

[74] Odunsi K., Wollman R.M., Ambrosone C.B., Hutson A., McCann S.E., Tammela J., Geisler J.P., Miller G., Sellers T., Cliby W. (2005). Detection of epithelial ovarian cancer using 1H-nmr-based metabonomics. Int. J. Cancer.

[75] Osl M., Drreiseitl S., Pfeifer B., Weinberger K., Klocker H., Bartsch G., Schafer G., Tilg B., Graber A. (2008). A new rule-based algorithm for identifying metabolic markers in prostate cancer using tandem mass spectrometry. Bioinformatics.

[76] Gao P. (2009). C-myc suppression of mir-23a/b enhances mitochondrial glutaminase expression and glutamine metabolism. Nature.

[77] Wang J., Yu L.F., Shen P., Wang S.F. (2009). Analysis of serum metabolome of patients with breast cancer by gas chromatography-mass spectrometry. Zhejiang Da Xue Bao Yi Xue Ban.

[78] Beger R., Schnackenberg L., Holland R., Li D., Dragan Y. (2006). Metabonomic models of human pancreatic cancer using 1d proton nmr spectra of lipids in plasma. Metabolomics.

[79] Yan S.K., Wei B.J., Lin Z.Y., Yang Y., Zhou Z.T., Zhang W.D. (2008). A metabonomic approach to the diagnosis of oral squamous cell carcinoma, oral clichen planus and oral leukoplakia. Oral Oncol..

[80] Kim R., Coates J., Bowles T., McNerney G., Sutcliffe J., Jung I., Gandour-Edwaeds R., Chuang F., Bold R., Kung H. (2009). Arginine deiminase as a novel therapy for prostate cancer induces autophary and caspase-independent apoptosis. Cancer Res..

[81] Nam H., Chung B.C., Kim Y., Lee K., Lee D. (2009). Combining tissue transcriptomics and urine metabolomics for breast cancer biomarker identification. Bioinformatics.

[82] Ganti S., Weiss R.H. (2011). Urine metabolomics for kidney cancer detection and biomarker discovery. Metabolomics.

[83] Poli D., Carbognani P., Corradi M., Goldoni M., Acampa O., Balbi B., Bianchi L., Rusca M., Mutti A. (2005). Exhaled volatile organic compounds in patients with non-small cell lung cancer: Cross sectional and nested short-term follow-up study. Respir. Res..

[84] Phillips M., Cataneo R.N., Ditkoff B.A., Fisher P., Greenberg J., Gunawardena R., Kwon C.S., Tietje O., Wong C. (2006). Prediction of breast cancer using volatile biomarkers in the breath. Breast Cancer Res..

[85] Nishiumi S., Shinohara M., Ikeda A., Yoshie T., Hatano N., Kakuyama S., Mizuno S., Sanuki T., Kutsumi H., Fukusaki E. (2010). Serum metabolomics as a novel diagnostic approach for pancreatic cancer. Metabolomics.

[86] Kim J.W., Dang C.V. (2006). Cancer’s molecular sweet tooth and the Warburg effect. Cancer Res..

[87] Tannock I.F., Rotin D. (1989). Acid ph in tumors and its potential for therapeutic exploitation. Cancer Res..

[88] Zamecnik P.C., Loftfield R.B., Stephenson M.L., Steele J.M. (1951). Studies on the carbohydrate and protein metabolism of the rat hepatoma. Cancer Res..

[89] Lv W., Yang T. (2012). Identification of possible biomarkers for breast cancer from free fatty acid profiles determined by GC/MS and multivariate statistical analysis. Clin. Biochem..

[90] Bhalla K., Hwang B.J., Dewi R.E., Ou L., Twaddel W., Fang H.-b., Vafai S.B., Vazquez F., Puigserver P., Boros L. (2011). Pgc1a promotes tumor growth by inducing gene expression programs supporting lipogenesis. Cancer Res..

[91] Dang C.V. (2010). Glutaminolysis: Supplying carbon or nitrogen or both for cancer cells?. Cell Cycle.

[92] Carracedo A., Cantley L.C., Pandolfi P.P. (2013). Cancer metabolism: Fatty acid oxidation in the limelight. Nat. Rev. Cancer.

[93] McKeehan W.L. (1982). Glycolysis, glutaminolysis and cell proliferation. Cell Biol. Int. Rep..

[94] Moreadith R.W., Lehninger A.L. (1984). The pathways of glutamate and glutamine oxidation by tumor cell mitochondria. Role of mitochondrial nad(p)+-dependent malic enzyme. J. Biol. Chem..

[95] Ben-Yoseph O., Badar-Goffer R.S., Morris P.G., Bachelard H.S. (1993). Glycerol 3-phosphate and lactate as indicators of the cerebral cytoplasmic redox state in severe and mild hypoxia respectively: A 13C- and 31P N. M. R. Study. Biochem. J..

[96] Griffiths J.R., Stubbs M. (2003). Opportunities for studying cancer by metabolomics: Preliminary observations on tumors deficient in hypoxia-inducible factor 1. Adv. Enzyme Regul..

[97] Struck W., Waszczuk-Jankowska M., Kaliszan R., Markuszewski M.J. (2011). The state-of-the-art determination of urinary nucleosides using chromatographic techniques “Hyphenated” With advanced bioinformatics methods. Anal. Bioanal. Chem..

[98] Zambonin C.G., Aresta A., Palmisano F., Specchia G., Liso V. (1999). Liquid chromatography determination of urinary 5-methyl-2'-deoxycytidine and psuedouridine as potential biomarkers for leukaemia. J. Pharm. Biomed. Anal..

[99] Sasco A.J., Rey F., Reynaud C., Bobin Y.L., Clavel M., Niveleau A. (1996). Breast cancer prognostic significance of some modified urinary nucleosides. Cancer Lett..

[100] Zheng Y.F., Kong H.W., Xiong J.H., Lv S., Xu G.W. (2005). Clinical significance and prognostic value of urinary nucleosides in breast cancer patients. Clin. Biochem..

[101] Woo H.M., Kim K.M., Choi M.H., Jung B.H., Lee J., Kong G., Nam S.J., Kim S., Bai S.W., Chung B.C. (2009). Mass spectrometry based metabolomic approaches in urinary biomarker study of women's cancers. Clin. Chem. Acta.

[102] Zheng Y.F., Yang J., Zhao X.J., Feng B., Kong H.W., Chen Y.J., Lv S., Zheng M.H., Xu G.W. (2005). Urinary nucleosides as biological markers for patients with colorectal cancer. World J. Gastroenterol..

[103] Yang J., Xu G., Zheng Y., Kong H., Pang T., Lv S., Yang Q. (2004). Diagnosis of liver cancer using hplc-based metabonomics avoiding false-positive result from hepatitis and hepatocirrhosis diseases. J. Chrom. B.

[104] Sreekumar A., Poisson L.M., Rajendiran T.M., Khan A.P., Cao Q., Yu J., Laxman B., Mehra R., Lonigro R.J., Li Y. (2009). Metabolomic profiles delineate potential role for sarcosine in prostate cancer progression. Nature.

[105] Olson R.E. (1951). Oxidation of C14-labeled carbohydrate intermediates in tumor and normal tissue. Cancer Res..

[106] Ackerstaff E., Pflug B.R., Nelson J.B., Bhujwalla Z.M. (2001). Detection of increased choline compounds with proton nuclear magnetic resonance spectroscopy subsequent to malignant transformation of human prostatic epithelial cells. Cancer Res..

[107] Glunde K., Jie C., Bhujwalla Z.M. (2004). Molecular causes of the aberrant choline phospholipid metabolism in breast cancer. Cancer Res..

[108] Hilvo M., Denkert C., Lehtinen L., Müller B., Brockmöller S., Seppänen-Laakso T., Budczies J., Bucher E., Yetukuri L., Castillo S. (2011). Novel theranostic opportunities offered by characterization of altered membrane lipid metabolism in breast cancer progression. Cancer Res..

[109] Dong J., Cai X., Zhao L., Xue X., Zou L., Zhang X., Liang X. (2010). Lysophosphatidylcholine profiling of plasma: Discrimination of isomers and discovery of lung cancer biomarkers. Metabolomics.

[110] Patterson A.D., Maurhofer O., Beyoğlu D., Lanz C., Krausz K.W., Pabst T., Gonzalez F.J., Dufour J.-F.o., Idle J.R. (2011). Aberrant lipid metabolism in hepatocellular carcinoma revealed by plasma metabolomics and lipid profiling. Cancer Res..

[111] Meleh M., Pozlep B., Mlakar A., Meden-Vrtovec H., Zupanic-Kralj L. (2007). Determination of serum lysophosphatidic acid as a potential biomarker for ovarian cancer. J. Chrom. B.

[112] Ringel M.D., Hayre N., Saito J., Saunier B., Schuppert F., Burch H., Bernet V., Burman K.D., Kohn L.D., Saji M. (2001). Overexpression and overactivation of akt in thyroid carcinoma. Cancer Res..

[113] Vivanco I., Sawyers C.L. (2002). The phosphatidylinositol 3-kinase-akt pathway in human cancer. Nat. Rev. Cancer.

[114] Fernandis A.Z., Wenk M.R. (2009). Lipid-based biomarkers for cancer. J. Chrom. B.

[115] Saddoughi S.A., Song P., Ogretmen B. (2008). Roles of bioactive sphingolipids and cancer biology and therapeutics. Subcell. Biochem..

[116] Nava V.E., Hobson J.P., Murthy S., Milstien S., Spiegel S. (2002). Sphingosine kinase type 1 promotes estrogen-dependent tumorigenesis of breast cancer mcf-7 cells. Exp. Cell Res..

[117] Sarkar S., Maceyka M., Hait N.C., Paugh S.W., Sankala H., Milstien S., Spiegel S. (2005). Sphingosine kinase 1 is required for migration, proliferation and survival of mcf-7 human breast cancer cells. FEBS Lett..

[118] Poullet J.-B., Martinez-Bisbal M., Valverde D., Monleon D., Celda B., Arus C., Van Huffel S. (2007). Quantification and classification of high-resolution magic angle spinning data for brain tumor diagnosis. Conf. Proc. IEEE Eng. Med. Biol. Soc..

[119] Tessem M.B. (2008). Evaluation of lactate and alanine as metabolic biomarkers of prostate cancer using 1H HR-MAS spectroscopy of biopsy tissues. J Magn. Reson. Med..

[120] Sitter B., Sonnewald U., Spraul M., Fjösne H.E., Gribbestad I.S. (2002). High-resolution magic angle spinning mrs of breast cancer tissue. NMR Biomed..

[121] Wilson M., Davies N.P., Brunder M.-A., McConville C., Grundy R.G., Peet A.C. (2009). High resolution magic angle spinning 1H NMR of childhood brain and nervous system tumors. Mol. Cancer.

[122] Somashekar B.S., Kamarajan P., Danciu T., Kapila Y.L., Chinnaiyan A.M., Rajendiran T.M., Ramamoorthy A. (2011). Magic angle spinning NMR-based metabolic profiling of head and neck squamous cell carcinoma tissues. J. Prot. Res..

[123] Stenman K., Stattin Pär., Stenlund H., Riklund K., Gröbner G., Bergh A. (2011). 1H hrmas nmr derived bio-markers related to tumor grade, tumor cell fraction, and cell proliferation in prostate tissue samples. Biomarker Insights.

[124] Cheng L.L., Burns M.A., Taylor J.L., He W., Halpern E.F., McDougal W.S., Wu C.-L. (2005). Metabolic characterization of human prostate cancer with tissue magnetic resonance spectroscopy. Cancer Res..

[125] Chan E.C.Y., Koh P.K., Mal M., Cheah P.Y., Eu K.W., Backshall A., Cavill R., Nicholson J.K., Keun H.C. (2008). Metabolic profiling of human colorectal cancer using high-resolution magic angle spinning nuclear magnetic resonance (HR-MAS NMR) spectroscopy and gas chromatography mass spectrometry (GC/MS). J. Prot. Res..

[126] Brindle K. (2008). New approaches for imaging tumour responses to treatment. Nat. Rev. Cancer.

[127] Friess H., Langhans J., Ebert M., Beger H.G., Stollfuss J., Reske S.N., Büchler M.W. (1995). Diagnosis of pancreatic cancer by 2 [18-F]-fluoro-2-deoxy-D-glucose positron emission tomography. Gut.

[128] Pöttgen C., Levegrün S., Theegarten D., Marnitz S., Grehl S., Pink R., Eberhardt W., Stamatis G., Gauler T., Antoch G. (2006). Value of 18f-fluoro-2-deoxy-d-glucose-positron emission tomography/computed tomography in non-small-cell lung cancer for prediction of pathologic response and times to relapse after neoadjuvant chemoradiotherapy. Clin. Cancer Res..

[129] Haddadin I.S., McIntosh A., Meisamy S., Corum C., Snyder A.L.S., Powell N.J., Nelson M.T., Yee D., Garwood M., Bolan P.J. (2009). Metabolite quantification and high-field mrs in breast cancer. NMR Biomed..

[130] Towner R.A., Foley L.M., Painter D.M. (2005). Hepatocarcinogenesis tumor grading correlated within vivo image-guided 1H-nmr spectroscopy in a rat model. Toxicol. Appl. Pharmacol..

[131] Carroll P., Coakley F., Kurhanewicz J. (2006). Magnetic resonance imaging and spectroscopy of prostate cancer. Rev. Urol..

[132] Yokota H., Guo J., Matoba M., Higashi K., Tonami H., Nagao Y. (2007). Lactate, choline, and creatine levels measured by vitro 1H-MRS as prognostic parameters in patients with non-small-cell lung cancer. J. Magn. Reson. Imaging.

[133] Law M. (2009). Advanced imaging techniques in brain tumors. Cancer Imaging.

[134] Türkbey B., Aras Ö., Karabulut N., Tuncay Turgut A., Akpinar E., Alibek S., Pang Y., Ertürk S., El Khouli R., Bluemke D. (2012). Diffusion-weighted mri for detecting and monitoring cancer: A review of current applications in body imaging. Diagn. Interv. Radiol..

[135] Türkbey B., Thomasson D., Bernardo M., Choyke P.L. (2010). The role of dynamic contrast-enhanced mri in cancer diagnosis and treatment. Diagn. Interv. Radiol..

[136] DeMartini W., Lehman C., Partridge S. (2008). Breast mri for cancer detection and characterization: A review of evidence-based clinical applications. Acad, Radiol..

[137] Warner E., Messersmith H., Causer P., Eisen A., Shumak R., Plewes D. (2008). Systematic review: Using magnetic resonance imaging to screen women at high risk for breast cancer. Ann. Intern. Med..

[138] Bartella L., Huang W. (2007). Proton (1H) MR spectroscopy of the breast. Radiographics.

[139] Dowling C., Bollen A.W., Noworolski S.M., McDermott M.W., Barbaro N.M., Day M.R., Henry R.G., Chang S.M., Dillon W.P., Nelson S.J. (2001). Preoperative proton mr spectroscopic imaging of brain tumors: Correlation with histopathologic analysis of resection specimens. Am. J. Neuroradiol..

[140] Seitz M., Shukla-Dave A., Bjartell A., Touijer K., Sciarra A., Bastian P.J., Stief C., Hricak H., Graser A. (2009). Functional magnetic resonance imaging in prostrate cancer. Europ. Urol..

[141] Alusta P., Im I., Pearce B.A., Beger R.D., Kretzer R.M., Buzatu D.A., Wilkes J.G. (2010). Improving proton mr spectroscopy of brain tissue for noninvasive diagnostics. J. Magn. Reson. Imaging.

[142] Elion G.B., Singer S., Hitchings G.H. (1954). Antagonists of nucleic acid derivatives: Viii. Synergism in combinations of biochemically related antimetabolites. J. Biol. Chem..

[143] Yauch R.L., Settleman J. (2012). Recent advances in pathway-targeted cancer drug therapies emerging from cancer genome analysis. Curr. Opin. Genet. Dev..

[144] Tennant D.A., Duran R.V., Gottlieb E. (2010). Targeting metabolic transformation for cancer therapy. Nat. Rev. Cancer.

[145] Weiss R.H., Kim K. (2012). Metabolomics in the study of kidney diseases. Nat. Rev. Nephrol..

[146] Bayet-Robert M., Morvan D., Chollet P., Barthomeuf C. (2010). Pharmacometabolomics of docetaxel-treated human mcf7 breast cancer cells provides evidence of varying cellular responses at high and low doses. Breast Cancer Res. Treat..

[147] Backshall A., Sharma R., Clarke S.J., Keun H.C. (2011). Pharmacometabonomic profiling as a predictor of toxicity in pateints with inoperable colorectal cancer treated with capecitabine. Clin. Cancer Res..

[148] Evelhoch J., Garwood M., Vigneron D., Knopp M., Sullivan D., Menkens A., Clarke L., Liu G. (2005). Expanding the use of magnetic resonance in the assessment of tumor response to therapy: Workshop report. Cancer Res..

[149] Zerhouni E.A., Sanders C.A., von Eschenbach A.C. (2007). The biomarkers consortium: Public and private sectors working in partnership to improve the public health. The Oncologist.

[150] Goodsaid F.M., Mendrick D.L. (2010). Translational medicine and the value of biomarker qualification. Sci. Transl. Med..

[151] Muirhead L.J., Kinross J., FitzMaurice T.S., Takats Z., Darzi A., Nicholson J.K. (2012). Surgical systems biology and personalized longitudinal phenotyping in critical care. Pers. Med..

[152] Nicholson J.K., Holmes E., Kinross J.M., Darzi A.W., Takats z., Lindon J.C. (2012). Metabolic phenotyping in clinical and surgical environments. Nature.

[153] Balog J., Szaniszlo T., Schaefer K.-C., Denes J., Lopata A., Godorhazy L., Szalay D., Balogh L., Sasi-Szabo L., Toth M. (2010). Identification of biological tissues by rapid evaporative ionization mass spectrometry. Anal. Chem..

[154] Oermann E.K., Wu J., Guan K.-L., Xiong Y. (2012). Alterations of metabolic genes and metabolites in cancer. Semin. Cell Dev. Biol..

[155] Singh A., Happel C., Manna S., Acquaah-Mensah G., Carratero J., Kumar S., Nasipuri P., Krausz K., Wakabayashi N., Ruby Dewi R. (2013). Nrf2 regulates mir-1 and mir-206 to drive tumorigenesis. J. Clin. Invest..

[156] Bertilsson H., Tessem M.-B., Flatberg A., Viset T., Gribbestad I., Angelsen A., Halgunset J. (2012). Changes in gene transcription underlying the aberrant citrate and choline metabolism in human prostate cancer samples. Clin. Cancer Res..

[157] Rantalainen M., Cloarec O., Beckonert O., Wilson I.D., Jackson D., Tonge R., Rowlinson R., Rayner S., Nickson J., Wilkinson R.W. (2006). Statistically integrated metabonomic—Proteomic studies on a human prostate cancer xenograft model in mice. J. Prot. Res..

[158] Ma Y., Zhang P., Wang F., Liu W., Yang J., Qin H. (2012). An integrated proteomics and metabolomics approach for defining oncofetal biomarkers in the colorectal cancer. Ann. Surg..

[159] Cuperlovic-Culf M., Ferguson D., Culf A., Morin P., Touaibia M. (2012). 1H nmr metabolomics analysis of glioblastoma subtypes: Correlation between metabolomics and gene expression characteristics. J. Biol. Chem..

[160] Eckhart A.D., Beebe K., Milburn M. (2012). Metabolomics as a key integrator for "Omic" advancement of personalized medicine and future therapies. Clin. Transl. Sci..

